# Optimizing diagnosis and management of ONFH, FNSF, and SIF

**DOI:** 10.1530/EOR-2025-0222

**Published:** 2026-07-01

**Authors:** Haley M Cox, Oluwafemi O Gbayisomore, Alejandro J Rivera-Vega, Nils Becker, Sina Habibollahi, Jack Porrino, Daniel Wiznia

**Affiliations:** ^1^ Yale School of Medicine, New Haven, Connecticut, USA; ^2^ Meharry Medical College, Nashville, Tennessee, USA; ^3^ Universidad Central del Caribe, Bayamón, Puerto Rico, USA; ^4^ Yale School of Medicine, Department of Orthopaedics & Rehabilitation, New Haven, Connecticut, USA; ^5^ Hannover Medical School, Hannover, Germany; ^6^ Yale New Haven Health Bridgeport Hospital, Department of Diagnostic Radiology, Bridgeport, Connecticut, USA; ^7^ Yale School of Medicine, Department of Radiology & Biomedical Imaging, New Haven, Connecticut, USA

**Keywords:** osteonecrosis of the femoral head, hip, femur, subchondral insufficiency fracture, femoral neck stress fracture, femoral neck stress reaction, avascular necrosis

## Abstract

- Osteonecrosis of the femoral head (ONFH), femoral neck stress fracture (FNSF), and subchondral insufficiency fracture (SIF) are distinct yet clinically overlapping causes of non-arthritic hip pain. All three conditions threaten femoral head viability via distinct pathophysiologic mechanisms and may lead to fracture displacement (FNSF) and irreversible collapse (ONFH, SIF, and displaced FNSF), ultimately necessitating arthroplasty if not identified and managed early.
- These conditions frequently present with groin pain and unremarkable radiographs in their early stages. Imaging may be normal or show generalized bone marrow edema. MRI is the most sensitive modality for detection; however, accurate diagnosis requires the integration of specific imaging features with the synthesis of the patient’s past medical history, including age, activity level, and bone quality.
- ONFH identified before collapse may be amenable to joint-preserving procedures, such as core decompression. FNSF requires urgent recognition and non-weight-bearing precautions to avoid displacement and vascular injury. SIF may be managed conservatively with protected weight-bearing and monitoring.
- Despite the expertise of experienced examiners and advanced imaging techniques, the initial diagnosis is frequently incorrect, and treatment options only become clear with timely follow-up and serial imaging.
- This review highlights diagnostic distinctions among ONFH, FNSF, and SIF, emphasizing clinical context, imaging-based differentiation, and appropriate management. Accurate early classification is crucial for guiding management decisions and preventing collapse of the femoral head or femoral neck displacement.

## Introduction

Non-arthritic hip pain in adults can result from a variety of underlying pathologies, including instability, dysplasia, impingement, neoplasms, high-energy fractures, and infections (1). Among the differentials, ONFH, FNSF, and SIF pose a particular diagnostic challenge due to the convergence of age-related risk (2, 3, 4, 5, 6), overlapping clinical presentations, and similar appearance of early imaging characteristics. While early magnetic resonance imaging (MRI) of FNSF approaches near-perfect sensitivity and specificity (7, 8), patients with misdiagnosis (9), delay to MRI (10, 11, 12, 13, 14), or negative MRI (15, 16) are at risk of severe complications, including progression to femoral head collapse, osteonecrosis and nonunion (Table 1) (17, 18). Misdiagnosis of ONFH and SIF is common, with rates of misdiagnosis that persist through end-stage surgical treatment ranging from 6% (19) to 50% (20) depending on the population and diagnostic pathway.

**Table 1:** Clinical pearls for differentiation of ONFH, FNSF, and SIF.

Distinct differences in patient profiles	
	ONFH typically occurs in patients aged 20–50; associated with corticosteroid use, alcohol, coagulopathies, systemic disease FNSF usually appears in young athletes/military (fatigue) or older adults with poor bone quality (insufficiency) SIF most often occurs in geriatric osteoporotic individuals (especially women), no discrete trauma
Imaging distinctions are critical	
	X-rays often normal early; MRI is gold standard but can be equivocal early on ONFH classic findings on MRI include serpiginous subchondral lesion, ‘double-line’ sign FNSF classic findings on MRI include a fracture line through the femoral neck with adjacent edema SIF classic findings on MRI include subchondral linear band with extensive bone marrow edema
Timely diagnosis prevents catastrophic outcomes	
	ONFH and SIF** →** collapse, secondary arthritis → THA FNSF → displacement → osteonecrosis or nonunion → THA
Management is disease- and stage-specific	
	ONFH pre-collapse treatment is core decompression ± biologics; post-collapse requires THA FNSF treatment differs; tension-sided FNSF requires fixation whereas stable compression-sided FNSF is managed with conservative care and monitoring SIF requires protected weight-bearing when pre-collapse; post-collapse requires THA

All three conditions threaten the viability of the femoral head via distinct pathophysiologic mechanisms and may lead to irreversible collapse and necessitate arthroplasty (Table 1) (17, 21, 22). Given that treatment strategies differ between these three pathologies (Table 1), establishing an early and accurate diagnosis is essential to optimizing outcomes (4, 21, 23, 24, 25, 26).

Initial radiographs are often non-discriminatory in early ONFH, FNSF, and SIF (Table 1) (26, 27, 28, 29). Even advanced imaging such as MRI or CT may yield subtle or equivocal findings in the early disease course (24, 25, 26). The limited early specificity of imaging across modalities reinforces the need for integrating imaging findings with clinical context, including patient history, physical exam, and risk factors (Table 1).

This review describes the clinical presentation and imaging findings of these three conditions to guide clinicians toward timely and accurate diagnosis and appropriate management.

## Methods

We conducted a narrative review of the literature on ONFH, FNSF, and SIF. The review began with established clinical practice guidelines, and the reference lists of these documents were used to identify additional studies. Expert input from the senior authors was used to supplement gaps in the literature and to contextualize conflicting findings. A structured PubMed search was performed using combinations of relevant keywords (e.g. ‘osteonecrosis’, ‘femoral neck stress fracture’, ‘subchondral insufficiency fracture’, ‘MRI’, ‘hip pain’), and a medical librarian was consulted to retrieve inaccessible articles. Additional sources were identified through backward citation and searches using OpenEvidence.

Articles were prioritized for inclusion if they reported original findings on clinical presentation, imaging features, management strategies, or outcomes. Secondary reviews were not prioritized, while case reports were included when they provided unique diagnostic or clinical insights. The selected articles were organized by diagnosis, and information was synthesized in representative tables to facilitate comparison and highlight useful distinctions among the three conditions.

## Pathophysiology, epidemiology, and risk factors

While ONFH, FNSF, and SIF are classically associated with specific patient populations, differentiation becomes most challenging when these entities present atraumatically in individuals who do not clearly fit traditional risk profiles. Diagnostic differentiation of ONFH from FNSF and SIF is complicated by both the increasing average age at diagnosis of ONFH (2, 3, 4) and emerging recognition of risk factors – low bone mineral density (osteopenia/osteoporosis), older age, female sex, and the absence of major trauma – which our review identifies as shared across these three entities (6, 30, 31, 32, 33). Accurate diagnostic prioritization and management in the interval before definitive diagnosis requires thoughtful evaluation of condition-specific risk factors, rooted in their underlying pathophysiologic mechanisms, and an emphasis on initial conservative care to mitigate the risk of the pathology progressing beyond the window for joint-preserving intervention.

### Osteonecrosis of the femoral head

ONFH is a progressive, ischemic disorder caused by compromised blood flow to the femoral head, leading to osteocyte death, subchondral collapse, and degenerative joint changes if untreated (34). It may result from trauma (such as hip dislocation or femoral neck fracture) or non-traumatic etiologies, which are more difficult to diagnose due to multifactorial and incompletely understood pathophysiology involving metabolic, genetic, and vascular factors (34, 35). While major non-traumatic ONFH risk factors are expanded in Supplementary Table 1 (see section on Supplementary materials given at the end of the article), it is worth emphasizing that corticosteroid use, alcohol abuse, and conditions compromising vascular health or systemic perfusion are recognized contributors to ONFH across any age group (35, 36, 37). Bilateral ONFH is present in up to 80% of patients with systemic risk factors and can develop asynchronously or synchronously (Supplementary Table 1) (25, 38). The condition most often affects middle-aged adults, with roughly equal sex distribution, and has an estimated incidence of 10,000–20,000 symptomatic cases annually in the US (Supplementary Table 1) (4, 25). This figure may underestimate true burden, as early-stage ONFH is often asymptomatic or under-recognized (20, 38, 39).

Given its progressive nature and clinical burden, several classification systems have been developed to guide staging and treatment (Supplementary Table 1) (40, 41, 42, 43). The ARCO classification is the most widely recommended system by international consensus because it subdivides pre-collapse ONFH into three types stratified by prognosis and risk of collapse, considering lesion size and location (28). The most recent revisions (2019 and 2021) define the stages outlined in Table 2 (44). This review uses the ARCO system for ONFH staging (Table 2).

**Table 2:** Association Research Circulation Osseous (ARCO) classification system for ONFH.

Stage	Description
Stage 0	Asymptomatic; normal imaging; rarely used because ONFH is typically not detected until imaging changes are present
Stage I	Normal radiographs; positive findings on MRI or bone scan (e.g. bone marrow edema, demarcated necrotic zone)
Stage II	Sclerosis, cysts, or osteopenia on radiographs; no collapse or flattening
Stage III	Subchondral collapse (crescent sign) or necrotic zone fracture
IIIA	Early collapse of <2 mm with subchondral fracture; differentiated from SIF based on the presence of a necrotic zone and the pattern of collapse
IIIB	Advanced collapse of >2 mm. SIF and FNSF rarely progress to this degree of collapse unless misdiagnosed or untreated
Stage IV	Femoral head flattening with joint space narrowing and acetabular involvement

### Femoral neck stress fracture

FNSFs develop when repetitive loading surpasses the bone’s capacity to remodel, ultimately leading to microstructural failure (33). FNSFs are broadly classified as fatigue fractures, which occur in healthy bone subjected to repetitive stress (e.g. military recruits, endurance athletes), and insufficiency fractures, which arise in weakened bone due to osteoporosis, vitamin D deficiency, disordered eating, or hormonal dysfunction (Supplementary Table 1) (21, 26, 33, 45). Female athletes with the female athlete triad are particularly vulnerable to both types (46, 47, 48). Fractures are further classified by location: tension-sided fractures occur on the superolateral femoral neck and carry a higher risk of displacement, while compression-sided fractures occur on the inferomedial neck and are typically more stable (Table 3) (49).

**Table 3:** Fullerton classification system for FNSF.

Stage	Description
Stage I	Compression-sided; stable
Stage II	Tension-sided; higher displacement risk
Stage III	Displaced; loss of cortical alignment, high complication risk (Fullerton & Snowdy (49))

### Subchondral insufficiency fracture

SIF of the femoral head results when physiologic stress is applied to structurally weakened subchondral bone, leading to microfractures that may progress to collapse if untreated (32, 50). SIF most commonly affects geriatric patients, particularly postmenopausal women with underlying osteoporosis (Supplementary Table 1) (30, 32). In these contexts, diminished bone mineral density weakens the subchondral plate, rendering it susceptible to linear insufficiency fractures, typically in the weight-bearing dome of the femoral head (51). No standardized classification system exists for SIF, but radiologists typically describe fracture morphology, edema pattern, and the presence or absence of collapse on MRI (Supplementary Table 1) (26). There is an evolving discussion surrounding transient osteoporosis of the hip (TOH), as recent literature suggests that many cases previously attributed to TOH are more accurately characterized as SIF when imaged with higher-resolution MRI (6, 52, 53, 54); unlike SIF, TOH follows a self-limited course (54, 55, 56).

## Clinical presentation

Clinically, ONFH, FNSF, and SIF commonly present with insidious-onset, progressive groin pain exacerbated by weight-bearing and improved by rest. Potential differences in onset, activity relationship, and severity raise or lower clinical suspicion for each (Supplementary Table 1) (21, 57, 58, 59). However, due to substantial overlap in clinical presentation, accurate diagnosis requires integration of patient history, risk factors, imaging findings, and longitudinal follow-up (30, 59, 60).

### Osteonecrosis of the femoral head

The pain pattern in symptomatic early ONFH – insidious onset, groin localization, and possible referral to the buttock or anterolateral thigh – is characteristic but not pathognomonic, and must be interpreted in the context of risk factors and imaging findings (Supplementary Table 1) (25). The pain is often aggravated by weight-bearing, and an antalgic gait or limp is frequently observed (Supplementary Table 1) (61). As the disease progresses and subchondral collapse occurs, patients may experience a sudden worsening of pain along with loss of hip motion (particularly internal rotation) (36, 62). Symptomatic ONFH patients may report prior ONFH in the contralateral hip or present with bilateral symptoms (58). In advanced ONFH, severe groin pain with joint dysfunction and collapse leads to significant disability (36, 62). Misdiagnosis or delayed diagnosis of ONFH is common due to the variable and subtle nature of early clinical presentation (20, 58).

### Femoral neck stress fracture

Patients with FNSF typically present with activity-related hip or groin pain that worsens with prolonged or intense physical activity and improves with rest (Supplementary Table 1) (21, 59, 63). The pain is rarely associated with a single antecedent traumatic event and often follows a history of escalating discomfort with weight-bearing activities (Supplementary Table 1) (21, 63). On examination, pain may be elicited with single-leg hop, axial loading, or stance tests (Supplementary Table 1) (59, 63). Depending on the location of the stress fracture, pain with passive internal rotation of the hip may or may not be prominent (Supplementary Table 1) (21, 63).

### Subchondral insufficiency fracture

Patients with a femoral head SIF typically describe an acute or subacute onset of groin pain without significant trauma, often severe enough to limit or prevent weight-bearing (Supplementary Table 1) (29, 52, 64). The pain is exacerbated by ambulation, and history may reveal a low-energy stumble or fall, increased activity in a patient with known osteoporotic bone, or current/recent pregnancy (Supplementary Table 1) (29, 52, 64).

## Imaging evaluation

Early diagnosis of ONFH, FNSF, and SIF is often delayed due to the limited sensitivity of plain radiographs, which often appear normal or nonspecific in the initial stages of disease (Supplementary Table 2) (25, 26, 65, 66, 67). While X-rays are useful for staging once structural changes have occurred, they have limited diagnostic utility in early disease (25, 26, 68). In patients with persistent hip pain and unremarkable X-rays, MRI is the preferred next step for diagnosis (25, 26, 65). MRI is particularly valuable in the early stages of disease, when clinical symptoms are present but radiographs are normal, and before structural collapse has occurred. CT may serve as a useful adjunct when MRI is contraindicated or when MRI findings are equivocal (24, 25, 26, 68, 69). CT is not recommended when MRI has provided a definitive diagnosis, due to its lower sensitivity for early marrow changes and the added radiation exposure (25, 26). Bone scan (bone scintigraphy) is generally nonspecific and not recommended for diagnosis (25, 26). Bone scan has limited utility in differentiating these diagnoses because both FNSF and SIF typically demonstrate focal increased radiotracer uptake at the fracture site, rendering the findings nonspecific (26, 70, 71). Bone scans may reveal photopenic zones in the femoral head due to infarct-related reduced perfusion during early ONFH, or in the acute or subacute phase following injuries such as femoral neck fracture (72). Imaging findings across all modalities depend on the stage of disease at the time of presentation (Supplementary Table 2). ONFH may occasionally be discovered incidentally on imaging obtained for unrelated concerns, underscoring the importance of careful image review in high-risk patients (73). Regular follow-up imaging may be necessary as some findings become more apparent over time (25, 26, 74).

## X-ray

In addition to standard anterior-posterior X-rays, frog leg lateral views of the hip should be acquired, which may provide subtle signs of ONFH or SIF (Supplementary Table 2) (25, 75, 76).

### Osteonecrosis of the femoral head

**Figure 1:**
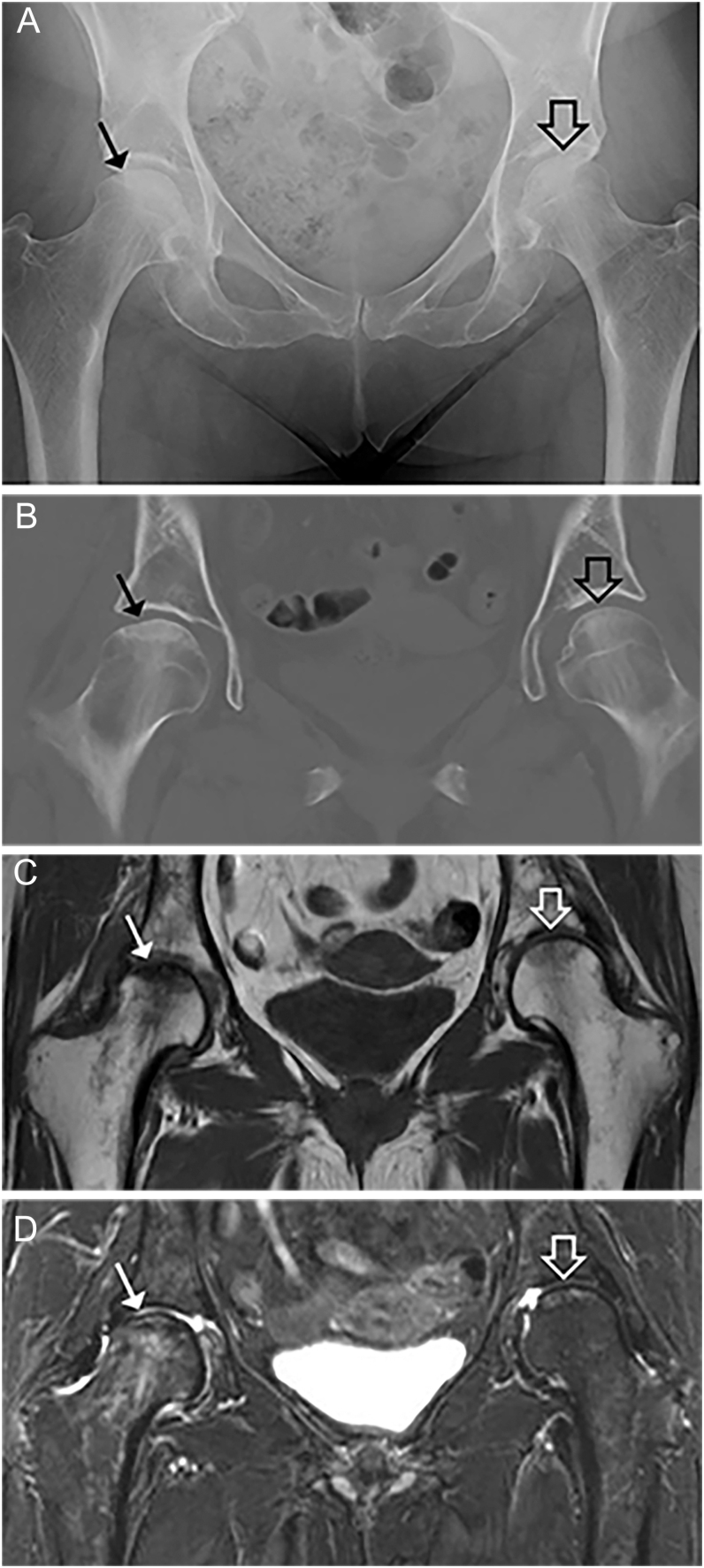
A 59-year-old female with hip pain – bilateral osteonecrosis of the femoral head. AP radiograph of the pelvis (A) and coronal CT of the pelvis (B) demonstrate subchondral sclerosis at the right femoral head with articular surface flattening (arrow), as well as subchondral sclerosis within the left femoral head (open arrow). Coronal T1 (C) and T2 fat-suppressed (D) MR images through the pelvis from MRI acquired one month later demonstrate serpiginous T1 hypointense and heterogeneous T2 signal abnormalities within the femoral heads with subchondral collapse on the right (arrow) but a normal spherical appearance of the left femoral head (open arrow).

As defined by ARCO, stage I X-rays of ONFH are normal (Supplementary Table 2) (25, 77, 66, 74). Stage II ONFH X-rays may show subtle findings or remain normal (Supplementary Table 2) (25, 77, 66, 74). Stage III lesions are characterized by femoral head collapse with a visible subchondral or necrotic zone fracture on plain radiographs (Fig. 1A) (25, 77, 66, 74). Stage IV lesions are characterized by secondary osteoarthritis changes (Supplementary Table 2).

### Femoral neck stress fracture

**Figure 2:**
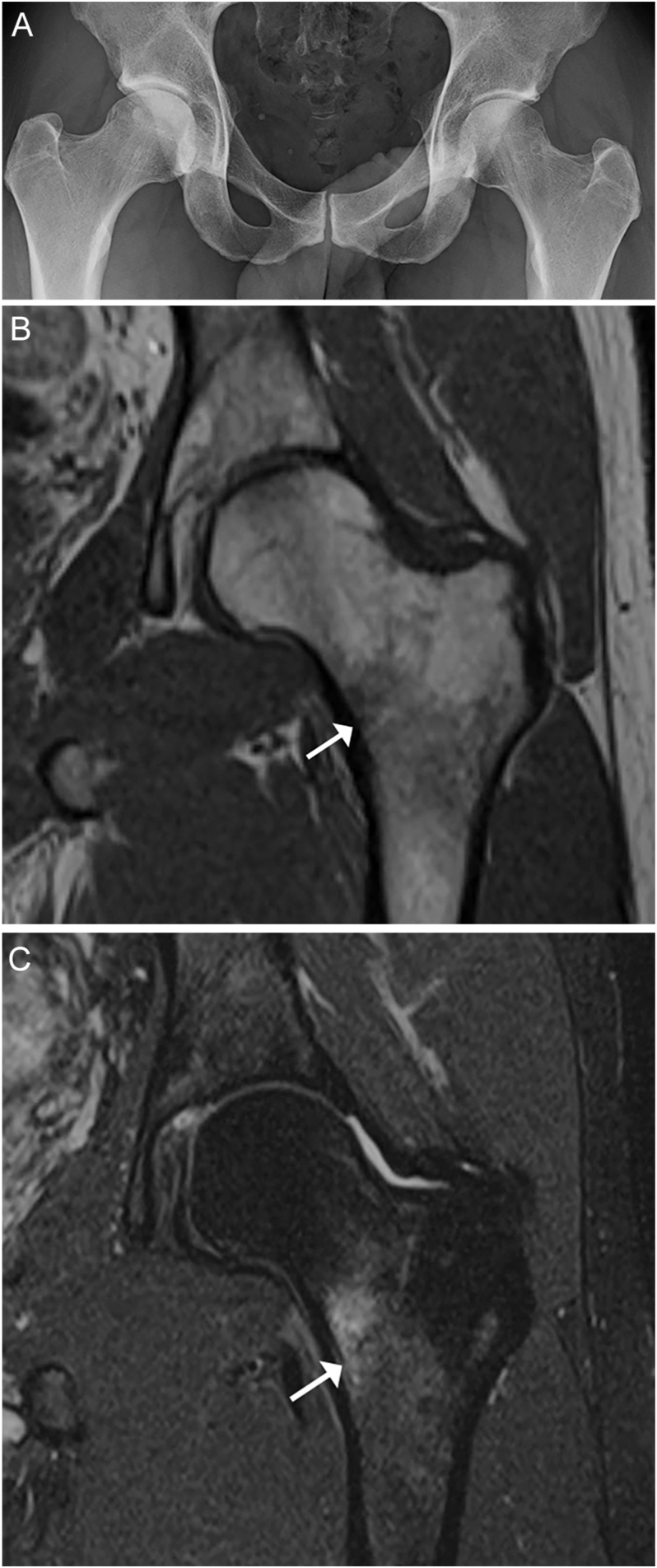
A 35-year-old male with recurrent left hip pain – femoral neck stress fracture. AP radiograph of the pelvis (A) demonstrates a normal appearance of the hips. Coronal T1 (B) and T2 fat-suppressed (C) MR images from MRI acquired one month later demonstrate bone marrow edema, which appears hyperintense on T2 and hypointense on T1 within the medial base of the left femoral neck, and with a subtle fracture plane, which appears most conspicuous on the T2-weighted image (arrows).

Early in the process, FNSF is not perceptible on X-ray (Fig. 2A). As this injury progresses, X-ray may reveal faint cortical irregularity, periosteal reaction, or subtle sclerosis at the femoral neck. Findings may eventually reveal a linear lucency representing the fracture line with or without callus formation (21, 33, 66).

### Subchondral insufficiency fracture

**Figure 3:**
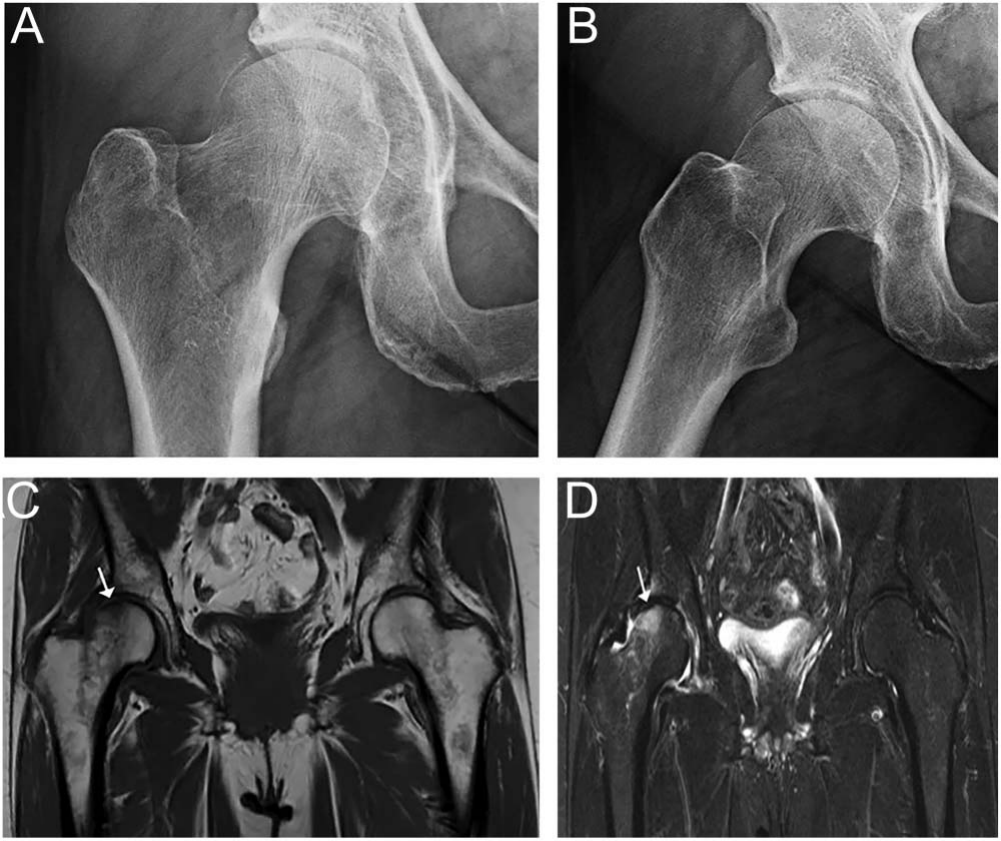
A 69-year-old female with right-sided hip pain – subchondral insufficiency fracture. AP (A) and lateral (B) radiographs of the right hip demonstrate a normal appearance of the right hip aside from regional chondrocalcinosis. Coronal T1 (C) and T2 fat-suppressed (D) MR images from MRI acquired 5 days later demonstrate marrow edema within the right femoral head with a subtle band of subchondral hypointense signal and articular surface flattening (arrows).

Pre-collapse X-ray imaging may appear normal or show signs of degeneration, such as subchondral sclerosis (Fig. 3A) (29, 78). After collapse, X-ray findings may include a crescent sign or subchondral flattening with surrounding subchondral sclerosis, closely resembling that of osteonecrosis (29, 30, 78).

## Magnetic resonance imaging

Among the available modalities, MRI is the most sensitive for highlighting early bone marrow and subchondral changes that are not visible on radiographs, and it offers high specificity in distinguishing among these pathologies as they progress (25, 26, 65, 67, 68, 79, 80). The American College of Radiology (ACR) Appropriateness Criteria recommend MRI in most clinical scenarios involving occult hip pain or suspected insufficiency fracture (25, 26). MRI is particularly valuable in the early stages of disease, when clinical symptoms are present but radiographs are normal, and before structural collapse has occurred. In such cases, MRI may reveal early pathology that cannot yet be definitively categorized as ONFH, FNSF, or SIF, but still warrants close monitoring or weight-bearing restrictions to prevent progression (25, 26). Bone marrow edema is best appreciated on fat-suppressed fluid-sensitive MRI sequences such as STIR or fat-suppressed T2-weighted images, where it appears as a region of high signal intensity (Supplementary Table 2) (81, 82, 83). A fracture line would be best visualized as a low-signal-intensity band on T1-weighted images (Supplementary Table 2) (84, 85). On fat-suppressed fluid-sensitive images, the fracture line remains low signal but is often surrounded by high-signal bone marrow edema (Supplementary Table 2) (84, 85).

### Osteonecrosis of the femoral head

MRI is the most sensitive imaging modality for detecting early-stage ONFH (stages I and II) (25, 66) and can identify the classic ‘double-line sign’ and delineate the extent of necrosis before structural collapse occurs, which is critical for prognosis and management (Supplementary Table 2) (65, 79). Stage I MRI shows a well-demarcated, subchondral, serpiginous band of low signal intensity on T1-weighted images, with corresponding high signal on T2-weighted images, with or without associated bone marrow edema (25, 66). Classic stage II MRI findings include the double line sign and a well-demarcated, subchondral, serpiginous band of low signal intensity on T1-weighted images, with corresponding high signal on T2-weighted images, with or without associated bone marrow edema (Supplementary Table 2) (25, 66, 86).

The modified Kerboul method, applied to MRI, is a validated and widely adopted tool for risk stratification in early-stage ONFH (87, 88). The Kerboul angle is calculated as the sum of the angular extent of the necrotic lesion measured on mid-coronal and mid-sagittal images, with a larger combined necrotic angle correlating with a higher risk of collapse and worse prognosis (28, 87). Although volumetric MRI techniques offer greater accuracy in estimating necrotic burden, the Kerboul angle remains clinically useful due to its simplicity and strong correlation with risk of femoral head collapse (25, 89). The ACR Appropriateness Criteria also identify lesion size and necrotic volume as key predictors of structural progression in osteonecrosis (25).

Stage III MRI findings include loss of femoral head sphericity and a linear subchondral low signal on both T1 (Fig. 1C) and T2-weighted (Fig. 1D) imaging with increased bone marrow edema, indicating the presence of a subchondral fracture described classically as a crescent sign (25, 66, 86). If left untreated, ONFH will progress to stage IV findings compatible with osteoarthritis (Supplementary Table 2) (22, 74).

### Femoral neck stress fracture

While not perceptible on X-ray, early FNSF manifests on MRI as bone marrow edema without a discrete fracture plane within the femoral neck (26). With progression, there is development of a linear hypointense fracture line on the superior lateral or inferior medial aspect of the femoral neck, often apparent on both T1 and fat-suppressed fluid-sensitive images, with regional bone marrow edema (Fig. 2B and C) (26).

### Subchondral insufficiency fracture

SIF is best appreciated on fat-suppressed fluid-sensitive MRI sequences as a thin, irregular, low-signal intensity band on T1-weighted images, typically convex toward the articular surface, with extensive surrounding bone marrow edema (BME) (Fig. 3C and D) (60, 67, 83). In early SIF, the band is nearer the subchondral plate and more subtle with BME prominent and often extending into the femoral neck (Supplementary Table 2). In contrast to early osteoarthritic changes, which may produce diffuse bone marrow lesions associated with cartilage loss, the discrete subchondral fracture line and disproportionate bone marrow edema of SIF are key distinguishing features on MRI (67). Radiographically, the absence of joint space narrowing favors SIF over OA (26). As SIF progresses, the low-signal band becomes more conspicuous and callus formation may develop overlying the fracture site, visible as a thickened subchondral plate on MRI (Supplementary Table 2) (60, 67, 90). Contrast-enhanced MRI shows enhancement of the segment proximal to the low-signal band in SIF, indicating viable bone as a key distinguishing feature from ONFH (27). This differs from the smooth, concave, and continuous bands observed in ONFH (85). Disease progression is indicated on T2-weighted images by subchondral intensity, more prominent bands with increased bone marrow edema, and overlying cartilage loss (83). Advanced stages correlate with collapse of the femoral head with secondary osteoarthritic changes (57).

## Computed tomography

CT provides an excellent visualization of cortical and subchondral bone integrity and can detect subtle fracture lines or early collapse that may be difficult to appreciate on MRI (26). In suspected ONFH or SIF, thin-slice CT may help confirm the presence of a subchondral fracture line (e.g. crescent sign) or reveal early collapse or subchondral sclerosis with greater detail (79, 91, 86). CT may also assist in distinguishing between collapse due to a fracture versus necrosis by evaluating fragmentation patterns and the presence of intra-articular calcified bodies (Supplementary Table 2) (86, 90, 92). In cases of ONFH where MRI is contraindicated, CT scan can be used to delineate the size, shape, and location of the necrotic lesion with high accuracy, identifying subtle subchondral fractures and bone resorption areas that are highly specific for early collapse (Supplementary Table 2) (25, 65, 93).

## Management and prognosis

Because ONFH, SIF, and FNSF have different etiologies, the management strategies and prognoses differ considerably (4, 21, 94). In all cases, early diagnosis and appropriate intervention are critical to prevent progression to femoral head collapse or femoral neck fracture displacement. A missed or delayed diagnosis can result in the need for more invasive treatments (such as total hip arthroplasty) and can adversely affect outcomes.

### Osteonecrosis of the femoral head

#### Stages I–II (pre-collapse)

In early-stage ONFH, treatment focuses on joint-sparing procedures designed to preserve the native femoral head (36). Core decompression (with or without adjunctive biologic therapies such as bone marrow aspirate concentrate or synthetic grafts) is a common first-line surgical treatment aimed at reducing intraosseous pressure and stimulating healing in early ONFH (4, 95). Hyperbaric oxygen therapy has demonstrated improvement in small studies (96, 97, 98, 99). Offloading and pharmacologic adjuncts (e.g. bisphosphonates or vasodilators) have been explored, but core decompression remains the standard (100, 101, 102).

#### Stage III (early collapse)

Stage III disease indicates structural compromise of the femoral head with a high risk of progression to joint collapse and secondary arthritis (77). If the femoral head has collapsed locally in a small region but the acetabulum remains intact, options include structural bone grafting or an osteotomy to redirect weight-bearing stress away from the necrotic segment (103, 104, 105). Partial resurfacing implants have a limited and highly selective role in the management of early-stage ONFH with clinical studies reporting rates of failure and conversion to total hip arthroplasty (THA) of up to 25% in the short- to mid-term (106). In cases with larger collapse areas, primary THA can be considered. The choice depends on the severity of the collapse, patient age, and activity demands (107).

#### Stage IV (advanced collapse with arthritis)

THA is usually the preferred treatment once there is significant femoral head collapse or secondary arthritis of the hip (107). Modern THA in ONFH patients yields excellent pain relief and functional outcomes, although younger patients should be counseled about the risks of future revision surgery (108, 109, 110).

### Femoral neck stress fracture

#### Compression-sided stress fractures (inferior femoral neck) without a clear fracture line

These fractures are at low risk of displacement and are typically managed conservatively (21). Non-weight-bearing or protected weight-bearing with crutches for 6–8 weeks is recommended, followed by a gradual return to weight-bearing as symptoms dictate (111). For long-term prevention, correction of any metabolic or nutritional deficiencies is recommended (112, 113, 114). Serial imaging is needed to ensure healing progress.

#### Tension-sided stress fractures (superior femoral neck) or displaced fractures

These fractures are at high risk of displacement and should be referred to an orthopedic surgeon for treatment (21, 45). Treatment includes strict non-weight-bearing with possible internal fixation due to the risk of displacement and development of osteonecrosis of the femoral head (21, 111). Percutaneous cannulated screw fixation or a dynamic hip screw is employed depending on fracture completeness (115).

### Subchondral insufficiency fracture

#### Pre-collapse insufficiency fracture

Non-surgical management is indicated for a non-displaced SIF before collapse occurs (26). This includes restricted weight-bearing (often touch-down weight-bearing with a walker), analgesia, and medications such as bisphosphonates or calcitonin (although evidence is limited) (94). Close follow-up with serial imaging is necessary. If collapse is avoided, some SIF lesions may stabilize and heal, and symptoms may resolve with conservative management.

#### Post-collapse femoral head insufficiency fracture

Once subchondral collapse has occurred, the condition typically progresses to end-stage osteoarthritis of the hip. In an older patient, total hip arthroplasty is the definitive treatment, as THA reliably alleviates pain and restores function (116). Unlike ONFH, there are no joint-preserving surgical options for post-collapse SIF, due to the poor bone quality and underlying osteoporosis (117, 118).

## Prognosis

Untreated ONFH and SIF share a poor prognosis, with a high likelihood of femoral head collapse leading to debilitating arthritis (25, 78, 119). The literature demonstrates that without intervention, between 30 and 77% of ONFH lesions will collapse within 2–3 years of symptom onset, depending on the size and location of the necrotic lesion (22, 65, 120, 121, 122). SIF has an even shorter timeline to collapse which is often within months of onset, given the acute structural compromise (83, 78). FNSF, if diagnosed and properly managed before displacement, carries an excellent prognosis (21). Compression-sided fractures typically heal without long-term sequelae, and tension-sided fractures, when internally fixed in a timely manner, often heal with preservation of the native hip (21). However, a displaced FNSF has a high risk of complications and may necessitate arthroplasty (21). Therefore, differentiating these diagnoses and instituting the correct treatment promptly is paramount to optimizing outcomes.

## Conclusion

Clinicians should rely on patient demographics, clinical history, symptom onset, and specific MRI findings to differentiate ONFH, FNSF, and SIF. Patients with previous corticosteroid use, alcohol abuse history, or underlying medical conditions compromising vascular or bone marrow health are understood to raise suspicion for ONFH (2, 3, 4). Rarely, SIF may progress to ONFH; however, this progression is uncommon compared to the classic risk factors discussed in this paper (56). In comparison, older patients with an acute onset of pain should raise concern for SIF, especially in the context of osteoporosis. The possibility of SIF should be investigated and followed up when findings include intense BME on MRI, especially in the presence of risk factors such as older age, osteopenia/osteoporosis, large joint effusion, or acetabular BME (6, 52). Gradually worsening, activity-related pain in athletes or military personnel strongly indicates FNSF (21). MRI characteristics, including distinct T1 and T2 signal patterns, are pivotal for early differentiation. In clinical scenarios where ONFH, FNSF, and SIF cannot be reliably distinguished based on demographics, clinical presentation, or MRI findings, an initially conservative approach with serial MRI and appropriate weight-bearing restrictions is recommended to monitor disease progression or resolution. Early and accurate diagnosis supports optimal management strategies, minimizes morbidity, and improves patient outcomes.

## Supplementary materials



## ICMJE Statement of Interest

The authors declare that there is no conflict of interest that could be perceived as prejudicing the impartiality of the work reported.

## Funding Statement

Research contributions by HMC in this publication were supported by the National Institute on Aging of the National Institutes of Health under Award Number T35AG049685. The content is solely the responsibility of the authors and does not necessarily represent the official views of the National Institute on Aging of the National Institutes of Health. Research contributions by NB were supported by the Deutsche Forschungsgemeinschaft (DFG, German Research Foundation).
