# Supplementary material for: Optimizing diagnosis and management of ONFH, FNSF, and SIF
Source: EFORT Open Rev. 2026 Jul 1;11(7):791–802. doi: 10.1530/EOR-2025-0222 (PMC13326886; doi:10.1530/EOR-2025-0222)
Supplement: Supplementary file 1 [file supplementary_materials.pdf]

Supplementary Table 1: Characteristic Clinical Features of ONFH, FNSF and SIF

**Osteonecrosis of the Femoral Head (ONFH)**	**Femoral Neck Stress Reaction and Fracture (FNSF)**	**Subchondral Insufficiency Fracture (SIF)**
	**Patient Profile**	
**ONFH, FNSF, and SIF** are most difficult to distinguish when they present atraumatically in patients between the ages of 15 and 50.		
**ONFH** is most often diagnosed between 20 and 50 years of age with a history of risk factors including high-dose corticosteroid use, excessive alcohol intake, anemias (sickle cell), and coagulation disorders<br><br>**__Traumatic ONFH__ can occur in any patient after femoral neck fracture or hip dislocation.*	**FNSF** is most often diagnosed in biphasic groups; between 20 and 40 years of age in active individuals (athletes, military recruits) for fatigue fractures; or adults over age 60 with osteopenia for insufficiency-type fractures.<br><br>Female gender and endocrine or nutritional factors (e.g. eating disorders) are risk factors for **fatigue FNSF**.<br><br><u>Patients may report:</u> recent increase in training intensity or a specific overuse pattern.	When diagnosed in non-geriatric patients, **SIF** may be associated with transient osteoporosis of the hip (i.e. late pregnancy or middle-aged men).<br><br>**SIF** is more commonly diagnosed in geriatric (over 60) patients, usually osteoporotic women.<br><br><u>Patients may report:</u> history of a low-energy stumble or fall, or increased activity on the background of osteoporotic bone.
	**Pain Characteristics**	
The early presentation of **ONFH**, **FNSF** and **SIF** (and many of the differentials) often have indistinguishable pain characteristics: insidious, progressive **groin pain** that **worsens with weight-bearing or activity** and **improves with rest or non-weightbearing.**		
**ONFH** presents with deep groin pain may radiate to the thigh, knee or buttock.<br><br>Most likely to present with broad radiation of pain.	**FNSF** presents with groin pain that is often well-localized early on; may radiate to anterior thigh or knee.	**SIF** presents with deep groin pain; can radiate to the medial thigh or buttock.<br><br>Most likely to have subtle radiation of pain with less distribution to the medial thigh or buttock than ONFH.

<table>
<tr><td>Exacerbated by internal rotation.</td><td>Exacerbated by activity or exercise; often rapidly relieved by rest.</td><td>Exacerbated severely by weight-bearing, often requiring assistive devices.</td></tr>
<tr><td>Unilateral and often bilateral

Contralateral hip may be asymptomatic before progression.</td><td>Typically unilateral but may be bilateral in high-risk patients.</td><td>Typically unilateral but may be bilateral in high-risk patients.</td></tr>
<tr><td colspan="3">In later stages when structural compromise or femoral head collapse has begun, the pain associated with ONFH, FNSF, and SIF converges in similarity both in intensity and character. Patients typically experience constant, severe groin pain that may radiate to the buttock, thigh, or knee and the pain often persists at rest and worsens with movement or weight-bearing. Nocturnal awakening may occur.</td></tr>
<tr><th colspan="3">Physical Exam</th></tr>
<tr><td colspan="3">Early physical exams for ONFH, FNSF and SIF are nearly indistinguishable, with normal or near-normal range of motion possibly provoking mild discomfort, positive leg roll and positive straight leg raise test, antalgic gait or subtle avoidance of full weight-bearing, and pain with active hip motion.</td></tr>
<tr><td>In early ONFH, physical exam is often normal or shows mild pain with internal rotation.</td><td>Early FNSF may show slightly more focal, reproducible pain with axial loading and tenderness to palpation in the groin.</td><td>In early SIF (pre-collapse), passive range of motion may be relatively maintained compared to pain level.

The patient may have a mild Trendelburg sign due to pain inhibition, but this is often non-specific</td></tr>
<tr><td colspan="3">In late stages, the physical exam for ONFH, FNSF and SIF converges to demonstrate painful and restricted hip motion in all planes, antalgic gait, or inability to bear weight. Physical exam indicators of femoral head collapse include limb shortening, a Trendelenburg gait, and severe pain with passive motion or axial loading.</td></tr>
<tr><th colspan="3">Classification System</th></tr>
<tr><td>Ficat and Arlet, Steinberg (University of Pennsylvania), Japanese Investigation Committee (JIC), Marcus Enneking

ARCO*: Stages ONFH by imaging and collapse (0-IV); includes MRI, X-ray, and bone scan findings.
*Used in this review</td><td>Fullerton: Classifies FNSF by MRI findings and fracture morphology (Types 1-3).</td><td>There is no universally accepted classification system for SIF, but radiologists typically report SIF identified on MRI by fracture line appearance, marrow edema pattern, and presence or absence of subchondral collapse.</td></tr>
</table>

Supplementary Table 2: Characteristic Imaging Findings in ONFH, FNSF, and SIF

**Osteonecrosis of the Femoral Head (ONFH) – see Figure 1**	**Femoral Neck Stress Fracture (FNSF) – see Figure 2**	**Subchondral Insufficiency Fracture (SIF) – see Figure 3**
**X-ray**		
Early plain X-ray are often normal in ONFH, FNSF, and early SIF. The ACR recommends including frog leg lateral views in the initial evaluation of clinically suspected ONFH.[1] This view enhances visualization of the femoral head contour and subchondral surface, which is critical for early identification of collapse and guiding appropriate management.		
**Stage I ONFH:** Normal.<br><br>**Stage II ONFH:** May show subtle findings such as patchy sclerosis and subchondral lucency without evidence of femoral head collapse.	**Early FNSF:** May be normal or show faint sclerosis. A compression-sided stress injury without a clear fracture line is usually not visible on X-ray. Tension-side incomplete fractures may appear as a faint linear radiolucency on the superior femoral neck in some cases.	**Early SIF:** Typically normal early on (insufficiency fractures are occult on initial X-ray). May see slight joint-space narrowing or vague subchondral lucency in some cases, but usually unremarkable.
**Stage III ONFH:** Lesions are characterized by the presence of a subchondral or necrotic zone fracture visible on X-ray, which is further divided into stages IIIA and IIIB. Subchondral lucency (crescent sign) and subchondral sclerosis are classic once collapse begins. Femoral head flattening/deformity in later stages.<br><br>→ Stage IIIA lesions show early collapse with femoral head depression $\leq$ 3mm.<br><br>→ Stage IIIB lesions show late collapse with femoral head depression >2mm.<br><br>**Stage IV ONFH:** Characterized by secondary osteoarthritic changes including joint space loss and osteophytes develop.[4,5]	**Advanced FNSF:** A cortical break or fracture line through the femoral neck becomes evident if the stress fracture propagates. Compression-side fractures may show callus or endosteal thickening. Displaced fractures are obvious with cortical disruption and angulation.	**Advanced SIF:** Once the insufficiency fracture progresses, radiographs show subchondral collapse and flattening of the femoral head, often with subchondral sclerosis that mimics ONFH (crescent sign can be seen). The key is that these changes occur in an older osteoporotic patient without typical ONFH risk factors.

Magnetic Resonance Imaging		
MRI is the standard of care for diagnosis of ONFH, FNSF, and SIF. T1-weighted images typically reveal areas of hypointensity, while T2 fat-suppressed sequences highlight hyperintense marrow changes, often with subtle subchondral lines or contour irregularities. These shared signal characteristics make MRI critical for identifying early-stage, nonspecific pathology before collapse.		
**Edema pattern** is typically localized to the necrotic segment and may spare adjacent bone.	**Edema pattern** is diffuse around a fracture line, often less intense than SIF.	**Edema Pattern** is diffuse, with extensive marrow edema of the femoral head and neck including the subchondral plate.
**Fracture line** is absent unless collapse has occurred.	**Fracture line** is a thin, sharply defined hypointense line **perpendicular** to the cortex.	**Fracture line** presents as an irregular hypointense line **parallel** to the articular surface in subchondral bone.
**Low-signal-intensity** band-like region in the superolateral femoral head is concave toward the articular surface.		**Low-signal-intensity** band-like region in the superolateral femoral head is convex toward the articular surface.

**Stage I ONFH:** Focal subchondral lesion, often subchondral; marrow signal alteration in anterosuperior femoral head with no collapse<br><br>**Stage II ONFH:** MRI demonstrates the classic "double line sign," or a peripheral, serpiginous band with inner bright (high signal) line and an outer dark (low signal) line on T2-weighted images.<br><br>**Stage III ONFH:** MRI demonstrates loss of femoral head sphericity and a linear subchondral low signal on both T1 and T2-weighted imaging with increased bone marrow edema, indicating the presence of a subchondral fracture described classically as a crescent sign.<br><br>**Stage IV ONFH:** MRI demonstrates advanced degenerative changes and joint space narrowing compatible with osteoarthritis.<br>**Advanced ONFH:** Subchondral collapse (crescent sign) with possible femoral head flattening, secondary osteoarthritis, joint effusion, and/or bone marrow edema	**Early FNSF:** Linear hypointense line on T1 and T2 (fracture line) with peri-fracture marrow edema often perpendicular to the femoral neck cortex<br><br>**Advanced FNSF:** Increased cortical disruption with widened fracture line, intensified marrow edema, displacement or loss of alignment and possible apparent cortical break.<br><br>Low-signal fracture line crossing the femoral neck with marked adjacent edema (T2 bright, T1 dark). Edema may extend into the shaft.	**Early SIF:** Curvilinear hypointense line on T1, paralleling subchondral surface with extensive ill-defined bone marrow edema of the femoral head and neck. The edema may cross the physis in skeletally immature patients.<br><br>**Advanced SIF:** Worsening marrow edema with progression of the subchondral fracture line, possible flattening or collapse of the femoral head with joint effusion and possible secondary osteoarthritic changes if chronic<br><br>Linear subchondral low-signal band (often irregular) with extensive surrounding marrow edema; band typically parallel to articular surface. After collapse, MRI resembles ONFH with sequestrum and secondary arthritic changes.
**Computed Tomography**		
CT excels at visualizing the cortical and subchondral bone integrity and can detect subtle fracture lines or early collapse that may be difficult to appreciate on MRI. Routine CT is not necessary if MRI has provided a clear diagnosis, due to the additional radiation and the superior sensitivity of MRI for early marrow changes.		
CT is not recommended for early detection of ONFH. It is useful to assess sequestrum and collapse; may show patchy sclerosis, cysts and fragmentation of subchondral bone in collapsed areas.	Can detect a hairline crack or periosteal reaction. For tension-side FNSF, CT helps visualize the fracture line in the superior cortex (only to be considered if MRI is unavailable).	Reveals fracture line through subchondral bone and depressed articular surface if collapse has occurred; subchondral sclerosis of fracture edges.
